# Pisa Syndrome in Parkinson’s Disease: Pathogenic Roles of Verticality Perception Deficits

**DOI:** 10.1038/s41598-018-20129-2

**Published:** 2018-01-29

**Authors:** Young Eun Huh, Kunhyun Kim, Won-Ho Chung, Jinyoung Youn, Seonwoo Kim, Jin Whan Cho

**Affiliations:** 1 Department of Neurology, CHA Bundang Medical Center, CHA University, Seongnam, 13496 Korea; 2 Department of Neurology, Sungkyunkwan University School of Medicine, Samsung Medical Center, Seoul, 06351 Korea; 3 Department of Otolaryngology, Head and Neck Surgery, Sungkyunkwan University School of Medicine, Samsung Medical Center, Seoul, 06351 Korea; 4 Statistics and Data Center, Samsung Medical Center, Seoul, 06351 Korea

## Abstract

We elucidated whether verticality misperception is associated with the generation of Pisa syndrome (PS) in patients with Parkinson’s disease (PD). To examine the heterogenous influence of verticality perception, we also identified the characteristics distinguishing between PD patients with PS who tilted toward the deviation of perceived verticality and those who did not. Subjective visual vertical (SVV) testing was performed in 54 PD patients with PS and 36 without PS to measure verticality perception. Other potential risk factors for PS were evaluated by assessing the asymmetry of motor symptoms, EMG activities of paraspinal muscles, bithermal caloric tests, back pain history, and Berg Balance Scale. Abnormal SVV (odds ratio (OR) 18.40, p = 0.006), postural imbalance (OR 0.71, p = 0.046), and unilateral EMG hyperactivity of paraspinal muscles (OR 39.62, p = 0.027) were independent contributors to PS. In subgroup analysis, EMG hyperactivity of paraspinal muscles contralateral to the leaning side and postural imbalance were associated with PD patients with PS who tilted toward the SVV deviation, whereas back pain was more frequent in those who did not. Verticality misperception is a potent risk factor for PS in PD and contributes differentially to PS depending on the congruence between its direction and PS direction, indicating distinct pathogenic roles.

## Introduction

Pisa syndrome (PS), characterized by a lateral tilt of the trunk that can be alleviated by passive movement or recumbent positioning, is a disabling postural deformity in patients with Parkinson’s disease (PD)^1–3^. Several hypotheses, including asymmetric basal ganglia output, impaired sensorimotor integration, musculoskeletal problems, and dystonia, have been proposed to explain the occurrence of PS, reflecting its complex pathophysiology^1–8^.

PD patients with PS also show abnormal perception of verticality in the roll plane^7,8^. To maintain an upright posture, sensory information from visual, vestibular, and somatosensory systems is centrally integrated to provide information to align the internal representation of the body axis with the earth-vertical^9^. Accordingly, damage to this integrative procedure may alter verticality perception, leading to postural misalignment. Indeed, impaired verticality perception, measured with subjective visual vertical (SVV) tests, is associated with lateral body tilts in several neurological conditions, including pusher syndrome and lateral medullary infarctions^10,11^. In these patients, body tilts are observed in the same direction of pathological perceived verticality, suggesting a primary pathogenic role of verticality misperception^10,11^. However, perceived verticality in PD patients with PS has been reported to deviate either towards or away from the side of body tilts^7,8^. Furthermore, other potential risk factors for PS have not been considered in previous reports^7,8^. Thus, the pathophysiological implications of verticality perception in PD patients with PS remain unclear, making appropriate management of PS more challenging.

In this study, we aimed to determine the pathogenic role of verticality perception to the generation of PS. Given the various involvement of verticality perception in PD patients with PS, we hypothesized that its role in pathogenesis of PS might be different depending on the congruence between the direction of perceived verticality and that of PS. To address this issue, we also examined the characteristics discriminating PD patients with PS who tilted to the same direction of perceived verticality from those who did not.

## Methods

### Study participants

Through the Movement Disorders Clinic of Samsung Medical Center, we recruited 90 patients fulfilling the UK PD Society’s Brain Bank criteria for idiopathic PD^12^. All included patients received at least one dopaminergic medication at stable and optimal doses over the preceding 4 weeks. Subjects who met these criteria were excluded: (1) postural deformities other than PS, including camptocormia, anterocollis, and retrocollis; (2) dyskinesia hindering adequate evaluation; (3) exposure to medications of potential relevance to PS, including antiemetics, neuroleptics, antidepressants, and central cholinergic inhibitors; (4) ocular misalignment or visual loss; (5) history or clinical signs of vestibular disorders, including spontaneous nystagmus or corrective saccades during head impulse testing; (6) signs of somatosensory disturbances, including decreased response of deep tendon reflex; (7) history of spinal surgery or major orthopedic problems disclosed by spine X-ray; (8) dementia; (9) neurosurgical intervention; (10) comorbid neurological disorders possibly affecting posture, such as stroke. All assessments were performed during the on-medication state.

Motor disability was scored using the Unified Parkinson Disease Rating Scale motor score (UPDRS-III). The dominant side and phenotype of motor symptoms at disease onset were recorded. The asymmetry of motor symptoms was defined as the sum of differences between each lateralized score of 20–26 items from UPDRS-III at the time of examinations^13,14^. Clinical postural stability was estimated using Berg Balance Scale (BBS), a 56-point scale in which lower scores indicate worse balance^15^. Global cognitive functions were tested using the Mini-Mental State Examination (MMSE). We calculated levodopa equivalent daily dose (LEDD)^16^ and classified treatment regimens according to the use of levodopa or dopamine agonists. The presence of back pain was documented when it lasted >12 months with a severity of ≥5 on a visual analogue scale and disturbed the patient’s activities of daily living.

**Figure 1:**
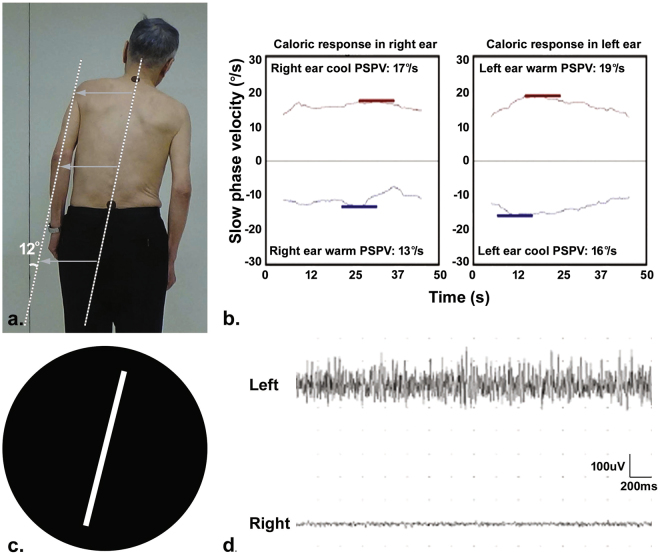
Clinical profiles in a PD-PS patient with ipsiversive subjective visual vertical. (**a**) Measurement of the degree of PS demonstrates trunk flexion to the right by approximately 12°. (**b**) The result of bithermal caloric test is normal. (**c**) Tests of subjective visual vertical (SVV) disclose abnormal SVV deviating by 14° to the right, ipsilateral to the leaning side. (**d**) EMG recording shows hyperactivity of the thoracolumbar paraspinal muscles on the left, contralateral to the leaning side. PSPV: peak slow phase velocity.

**Figure 2:**
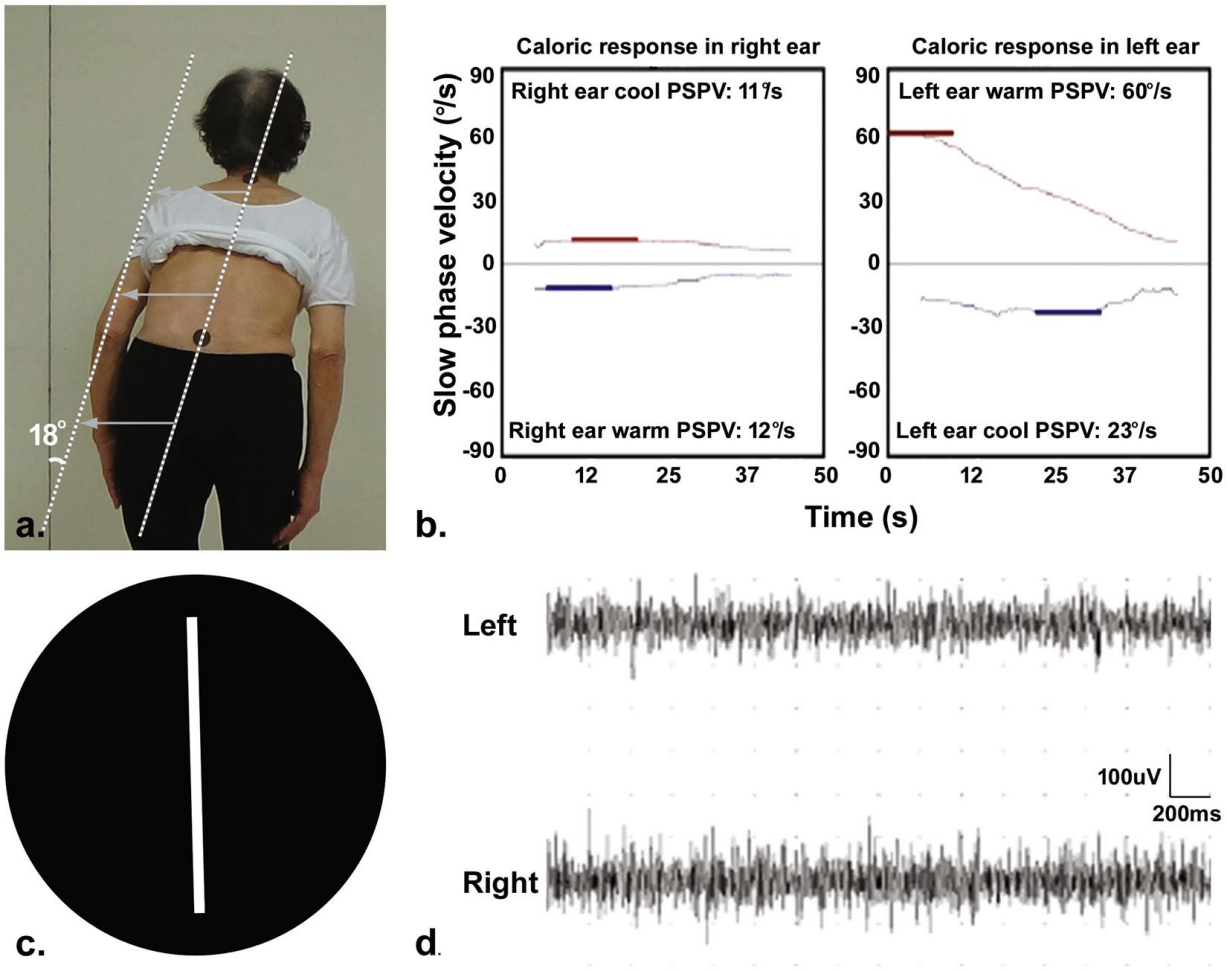
Clinical profiles in a PD-PS patient with ipsilateral canal paresis. (**a**) Measurement of the degree of PS demonstrates trunk flexion to the right by approximately 18°. (**b**) Bithermal caloric test discloses canal paresis (57%) on the right, ipsilateral to the leaning side. (**c**) The result of subjective visual vertical tests is normal (1.5° to the left). (**d**) EMG recording shows hyperactivity of the thoracolumbar paraspinal muscles on both sides. PSPV: peak slow phase velocity.

Patients were assigned to the PD with PS (PD-PS) group if they exhibited a lateral tilt of the trunk ≥10° that was reversed while supine or passively mobilized^1,2^. We determined the degree of a lateral trunk tilt while the subject was standing by measuring the angle between a vertical line on the wall and an imaginary line passing through markers placed at C7 and L4 (Figs 1 and 2). A body tilt was documented using a digital camera positioned 2 m behind the subject at a height of 1 m. PD-PS and PD patients without PS (PD-noPS) were matched for age and on Hoehn and Yahr stage ranging from 2 to 3.

### Standard protocol approvals, registration, and patient consents

Written informed consent for both study participants and publication of identifying images/information in an online open-access publication was obtained from each participant prior to the study. This study protocol was in accordance with Declaration of Helsinki and approved by the Institutional Review Board of Samsung Medical Center.

### Subjective visual vertical testing

The ability to perceive verticality was assessed using SVV tests^17^. Patients sat in a dark room viewing a rod (8.4-cm long, 0.4-cm wide) on a liquid-crystal display monitor positioned 1 m from their eyes. The rod was viewed through a hole (15 cm in diameter) in a black panel positioned in front of the monitor to prevent visual inference of verticality from the surroundings. Patients were instructed to sit upright, keeping their head upright. The rod was randomly presented at various angles from the vertical. Patients were asked to verbally instruct the examiner to move the rod into a vertical position. The examiner, who was unaware of the rod’s position, rotated the rod using a computer mouse until the patients said the rod was exactly vertical. The SVV value was determined by averaging the results from 10 trials under binocular viewing conditions. SVV was considered abnormal when it exceeded the normal range (mean ± 2 SD = −3.0 to 3.0°) obtained from 40 healthy control (20 men; mean age, 57.2 years [range, 45–72]). An abnormal SVV in the same direction of lateral trunk flexion was designated as ipsiversive SVV.

### EMG analysis

We performed multi-channel EMG recordings using a conventional EMG machine (Viking IV, Nicolet Biomedicals, Madison, USA). EMG activity from bilateral paraspinal thoracolumbar (T12-L1)^5^ muscles were recorded with silver-silver chloride surface electrodes in the static position while patients stood with a relaxed posture. EMG signals were amplified and bandpass-filtered between 100 and 2000 Hz. EMG recordings were sampled for 60 seconds in 15 consecutive 4-second sweeps. EMG hyperactivity was defined as the presence of tonic EMG discharges, indicating sustained muscle contraction.

### Bithermal caloric tests

Caloric stimuli were provided by irrigating each ear with cold (30.5 °C) and warm (43.5 °C) air. Asymmetries of vestibular response between ears were calculated using Jongkee’s formula and response asymmetries >25% were considered as canal paresis (CP)^18^. Unilateral CP on the same side of lateral trunk flexion was designated as ipsilateral CP.

### Statistical analysis

SAS version 9.4 (SAS Institute, Cary, NC) and R 3.3.2 (Vienna, Austria; http://www.R-project.org/) were used for statistical analysis. The significance level was set at 0.05. To assess the association between potentially relevant factors and the presence of PS, we conducted univariable and multivariable logistic regression analyses using Firth’s penalized maximum likelihood estimation method. For multivariable analysis, we used variables with p < 0.2 in univariable analysis. Variables with a high level of multi-collinearity (variance inflation factors ≥4) were excluded from the multivariable model. To investigate the different influence of SVV on PS depending on the congruence between the direction of SVV and that of PS, we further divided PD-PS patient into two groups, PD-PS with ipsiversive SVV and PD-PS without ipsiversive SVV, then repeated the univariable and multivariable analyses.

### Data availability

The datasets generated during and analyzed during the current study are not publicly available due to patient confidentiality but are available from the corresponding author on reasonable request.

## Results

### Association between abnormal SVV and the presence of PS

**Figure 3:**
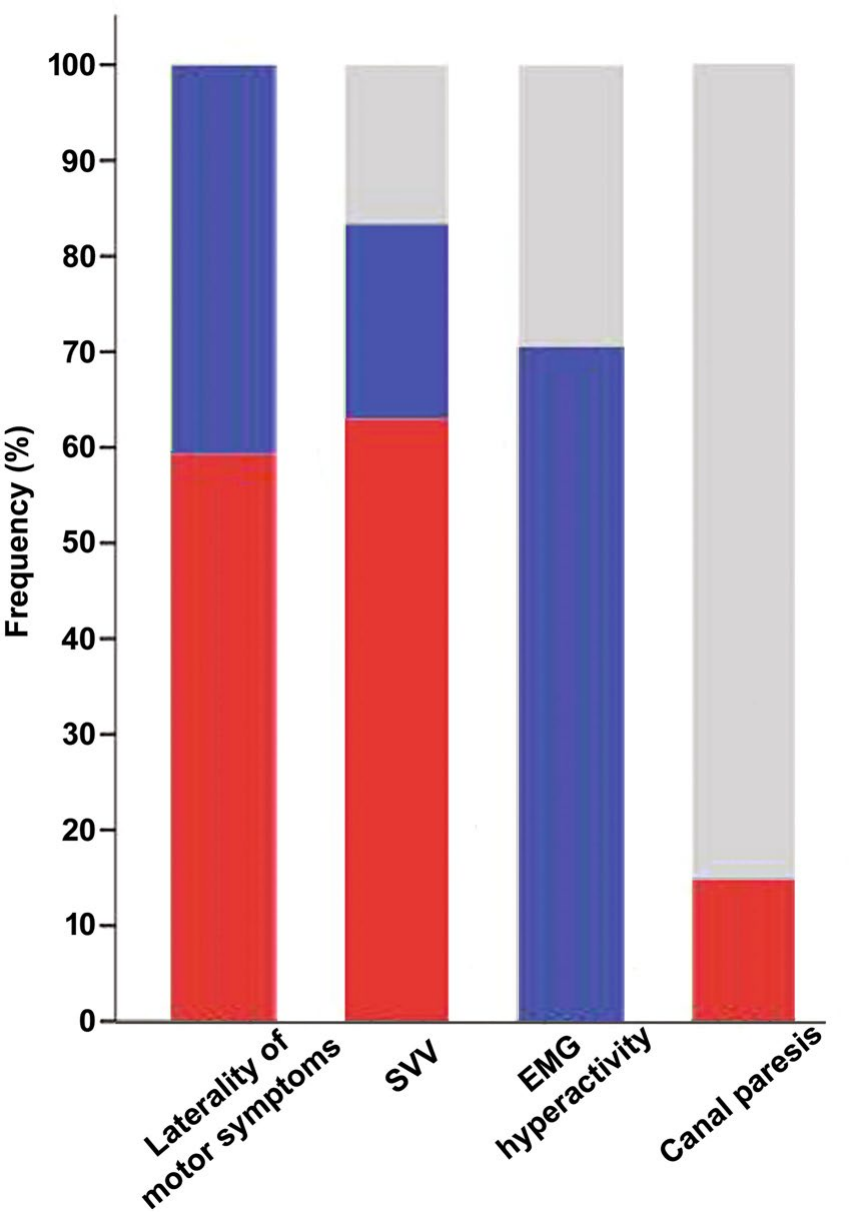
Distribution of variables with directionality in PD-PS patients. Color bars represent the frequencies of PD-PS patients for each variable with directionality, including laterality of motor symptoms, subjective visual vertical (SVV), EMG hyperactivity, or canal paresis, respectively. Red color illustrates the frequency of PD-PS patients tilting to the less affected side, with ipsiversive SVV, ipsilateral EMG hyperactivity, or ipsilateral canal paresis. Blue color depicts the frequency of PD-PS patients tilting to the more affected side, with contraversive SVV, contralateral EMG hyperactivity, or contralateral canal paresis. Gray color represents the frequency of PD-PS patients who exhibit normal SVV, bilateral EMG hyperactivity, or no canal paresis.

Demographics and clinical measurements are presented in Table 1. In our PD-PS, the lateral flexion of the trunk ranged from 12 to 22 degrees and increased with the degree of SVV tilt (r = 0.485, p < 0.001). The onset of postural deformity in PD-PS patients was insidious, lasting more than 3 months. Forty-five of PD-PS patients (83.3%) exhibited abnormal SVV (Table 1) and 34 of them (63%) deviated to the leaning side (Figs 1 and 3), whereas only one PD-noPS patient had an abnormal SVV. Unilateral EMG hyperactivity of paraspinal muscles was only observed in PD-PS patients (Table 1), which was uniformly contralateral to the leaning side (Fig. 3). The frequency of unilateral CP did not differ between PD-PS and PD-noPS patients (Table 1), and always ipsilateral when present in PD-PS patients (n = 8, 14.8%; Figs 2 and 3). The frequency of asymmetry of motor symptoms did not differ between PD-PS and PD-noPS patients, but 32 of PD-PS patients (59.3%) tilted toward the less affected hemibody (Fig. 3). Chronic back pain was more common and postural imbalance more severe in PD-PS patients compared with PD-noPS patients (Table 1). There were no differences in age, gender, disease duration or severity, motor subtype, dominant side of motor symptoms, LEDD, treatment regimens, body mass index (BMI), or MMSE between groups. Multivariable analysis revealed that abnormal SVV, postural imbalance, and unilateral EMG hyperactivity of paraspinal muscles were independently associated with PS.

**Table 1:** Univariable and multivariable analysis of risk factors associated with Pisa syndrome in patients with Parkinson’s disease.

	PD-PS (n = 54)	PD-noPS (n = 36)	Univariable analysis*			Multivariable analysis†		
			OR	95% CI	p value	OR	95% CI	p value
Age (years)	66.4 ± 6.4	66.9 ± 5.7	0.99	0.92 to1.06	0.761	—	—	—
Male gender (%)	33 (61.1)	18 (50)	1.56	0.67 to 3.65	0.307	—	—	—
Disease duration (years)	7.9 ± 3.4	8.0 ± 3.5	1.00	0.88 to1.13	0.959	—	—	—
Hoehn and Yahr stage	2.2 ± 0.3	2.3 ± 0.3	0.70	0.20 to 2.53	0.589	—	—	—
UPDRS-III	22.8 ± 5.8	22.9 ± 5.5	1.00	0.93 to 1.08	0.954	—	—	—
PD motor subtype (PIGD/TD)	23/31	18/18	0.75	0.32 to1.74	0.498	—	—	—
Abnormal SVV (%)	45 (83.3)	1 (2.8)	113.36	18.80 to 683.38	<0.001	18.40	2.33 to 145.00	0.006
Asymmetry of motor symptoms	5.5 ± 2.6	4.8 ± 1.3	1.17	0.95 to 1.43	0.137	—	—	—
Dominant side of motor symptoms (R/L)	32/22	18/18	1.44	0.62 to 3.37	0.396	—	—	—
Canal paresis (%)								
Unilateral	8 (14.8)	4 (11.1)	1.32	0.37 to 4.71	0.669	—	—	—
No	46 (85.2)	17 (85.0)	—	—	—	—	—	—
EMG patterns of paraspinal muscles (%)								
Unilateral hyperactivity	38 (70.4)	0	170.34	9.50 to 3054.86	0.001	39.62	1.52 to 1035.36	0.027
Bilateral hyperactivity	16 (29.6)	36 (100.0)	—	—	—	—	—	—
Back pain (%)	25 (46.3)	7 (19.4)	3.40	1.28 to 9.00	0.014	3.36	0.60 to 18.71	0.167
BBS	49.4±3.1	53.6 ± 1.5	0.48	0.35 to 0.66	<0.001	0.71	0.50 to 0.99	0.046
LEDD (mg)	593.7±218.0	604.0 ± 222.0	1.00	0.99 to 1.00	0.826	—	—	—
Treatment regimen (%)								
Levodopa + dopamine agonist^‡^	44 (81.5)	27 (75.0)	—	—	—	—	—	—
Levodopa	4 (7.4)	6 (16.7)	0.43	0.09 to 2.00	0.434	—	—	—
Dopamine agonist	6 (11.1)	3 (8.3)	1.15	0.22 to 6.03	1.000	—	—	—
BMI (kg/m^2^)	24.1 ± 2.6	23.4 ± 3.2	1.10	0.94 to 1.28	0.239	—	—	—
MMSE	28.0 ± 1.9	27.9 ± 1.9	1.03	0.82 to 1.29	0.795	—	—	—
Education (years)	11.6 ± 3.6	11.6 ± 3.9	1.00	0.90 to 1.12	0.962	—	—	—

*p values and 95% CI were corrected using Bonferroni’s correction for multiple tests. ^†^Variables with p < 0.2 in univariable analysis were included in multivariable analysis. ^‡^Reference. BBS: Berg Balance Scale, BMI: body mass index, LEDD: levodopa equivalent daily dose, MMSE: Mini-Mental State Examination, PIGD: postural instability and gait disturbance, SVV: subjective visual vertical, TD: tremor dominant, UPDRS-III: Unified Parkinson Disease Rating Scale motor score.

### Differences between PD-PS patients with and without ipsiversive SVV

Subgroup analysis showed that EMG hyperactivity of paraspinal muscles contralateral to the leaning side was more frequent in PD-PS patients with ipsiversive SVV than in PD-PS patients without ipsiversive SVV (Table 2 and Fig. 1). PD-PS patients with ipsiversive SVV also had more difficulty maintaining postural balance than those without ipsiversive SVV. Compared with PD-PS patients with ipsiversive SVV, chronic back pain was more common in PD-PS patients without ipsiversive SVV. The two groups did not differ in age, gender, disease duration and severity, motor subtype, degree and direction of PS, degree of SVV, asymmetry and dominant side of motor symptoms, frequency of PS tilting to the less affected hemibody, frequency of ipsilateral CP, LEDD, treatment regimens, BMI, and MMSE. In multivariable analysis, poor postural balance and EMG hyperactivity of paraspinal muscles contralateral to the leaning side were independently associated with PD-PS with ipsiversive SVV, whereas chronic back pain was negatively associated with PD-PS with ipsiversive SVV.

**Table 2:** Univariable and multivariable analysis of risk factors associated with ipsiversive SVV in PD-PS patients.

	PD-PS with ipsiversive SVV (n=34)	PD-PS without ipsiversive SVV (n=34)	Univariable analysis*			Multivariable analysis†		
			OR	95% CI	p value	OR	95% CI	p value
Age (years)	66.74 ± 5.4	65.95 ± 8.0	1.02	0.93 to 1.11	0.681	—	—	—
Male gender (%)	20 (58.8)	13 (65.0)	0.79	0.25 to 2.46	0.679	—	—	—
Disease duration (years)	7.7 ± 3.0	8.4 ± 4.1	0.94	0.80 to 1.11	0.462	—	—	—
Hoehn and Yahr stage	2.2 ± 0.4	2.2 ± 0.3	1.65	0.29 to 9.42	0.574	—	—	—
UPDRS-III	22.7 ± 6.4	23.1 ± 4.6	0.99	0.90 to 1.09	0.790	—	—	—
PD motor subtype (PIGD/TD)	15/19	8/12	1.17	0.38 to 3.58	0.785	—	—	—
Degree of PS (°)	15.4 ± 3.26	14.8 ± 2.75	1.07	0.88 to 1.29	0.513	—	—	—
Direction of PS (R/L)	19/15	14/6	0.56	0.18 to 1.81	0.335	—	—	—
Degree of SVV (°)	6.5 ± 3.4	5.1 ± 4.7	1.09	0.93 to 1.27	0.284	—	—	—
Asymmetry of motor symptoms	5.6 ± 2.8	5.3 ± 2.3	1.05	0.84 to 1.30	0.689	—	—	—
PS tilting to the less affected hemibody (%)	21 (61.8)	11 (55.0)	1.32	0.43 to 4.02	0.630	—	—	—
Dominant side of motor symptoms (R/L)	22/12	10/10	1.80	0.59 to 5.52	0.304	—	—	—
Canal paresis (%)								
Ipsilateral	4 (11.8)	4 (20.0)	0.54	0.12 to 2.46	0.426	—	—	—
No	30 (88.2)	16 (80.0)	—	—	—	—	—	—
EMG patterns of paraspinal muscles (%)								
Contralateral hyperactivity	28 (82.4)	10 (50.0)	4.38	1.27 to 15.11	0.019	5.76	1.04 to 31.95	0.045
Bilateral hyperactivity	6 (17.6)	10 (50.0)	—	—	—	—	—	—
Back pain (%)	11 (32.3)	14 (70.0)	0.22	0.07 to 0.72	0.012	0.21	0.05 to 0.95	0.043
BBS	48.1 ± 2.4	51.6 ± 2.8	0.63	0.48 to 0.82	0.001	0.66	0.50 to 0.88	0.005
LEDD (mg)	569.3 ± 197.5	635.1 ± 248.8	0.99	0.99 to 1.00	0.310	—	—	—
Treatment regimen (%)								
Levodopa + dopamine agonist	27 (79.4)	17 (85.0)	1.00^‡^	—	—	—	—	—
Levodopa	3 (8.8)	3 (15.0)	0.64	0.09 to 4.50	1.000	—	—	—
Dopamine agonist	4 (11.8)	0	5.72	0.13 to 255.53	0.607	—	—	—
BMI (kg/m^2^)	24.4 ± 2.7	23.7 ± 2.6	1.10	0.89 to 1.36	0.371	—	—	—
MMSE	27.6 ± 2.0	28.6 ± 1.5	0.74	0.52 to 1.06	0.101	0.84	0.53 to 1.34	0.468
Education (years)	11.4 ± 3.7	12 ± 3.5	0.95	0.82 to 1.11	0.546	—	—	—

*p values and 95% CI were corrected using Bonferroni’s correction for multiple tests. ^†^Variables with p < 0.2 in univariable analysis were included in multivariable analysis. ^‡^Reference. BBS: Berg Balance Scale, BMI: body mass index, LEDD: levodopa equivalent daily dose, MMSE: Mini-Mental State Examination, PIGD: postural instability and gait disturbance, SVV: subjective visual vertical, TD: tremor dominant, UPDRS-III: Unified Parkinson Disease Rating Scale motor score.

## Discussion

A vast body of literature has proposed numerous hypotheses regarding the pathophysiology of PS^1–3^. However, few studies systematically assessed perceptual alteration of verticality in PD patients with PS^7,8^, although defected body schema (i.e., misperception of the body in space) plays a key role in postural control deficits in PD^19^. Furthermore, other potential risk factors for PS have rarely been considered in previous reports, providing limited evidence for a pathogenic role of verticality misperception in PS. In fact, PD patients with PS have shown variable responses to treatments targeting a single pathophysiology of PS^20^. Accordingly, defining distinct properties correlated with patterns of verticality perception in PD patients with PS may lead to new therapeutic approaches for PS. This also helps promote the efficacy of those therapies by precisely identifying patients who will benefit from them. Our study shed some lights on these issues by confirming the independent pathogenic role of verticality misperception to PS and delineating the clinical heterogeneity of verticality perception in PD patients with PS.

We found that disruptive verticality perception in the roll plane was an important risk factor for PS. Our SVV adjustment tasks depend primarily on gravitational inputs from vestibular otolithic system and somatosensory graviceptors^21,22^. Accordingly, abnormal SVV in our study may result from impaired integration of gravitational signals from these two sensory systems. Thus, our finding is comparable to a recent study which demonstrated veering while walking with eyes closed, representing unbalanced processing of vestibular or proprioceptive feedbacks, as an independent risk factor for PS^14^. Our result is also consistent with a recent hypothesis which emphasizes sensory integration failure and consequent misperception of body schema as key factors leading to PS^1,2^. Additionally, pathomechanism of perceptual deficits in gravitational vertical can explain the marked improvement of PS in the supine position, in which the influence of gravity lessens^1,2^. As most of our PD patients did not exhibit sensory loss affecting peripheral apparatus, abnormal SVV may stem from dysfunction in central graviceptive network, in agreement with previous reports^23–25^. Central graviceptive pathway travels from the vestibular nuclei, either crosses or uncrosses in the brainstem, then reaches higher cortical and subcortical structures, engaging the posterior lateral thalamus, basal ganglia, insular cortex, inferior frontal gyrus, superior temporal gyrus, and inferior parietal lobule^17,26^. Damage to these structures is commonly associated with postural and locomotor deficits in PD patients by interrupting integration of multisensory information for ensuring spatial orientation^19,27,28^. Accordingly, asymmetric involvement of PD pathology might cause biased signal processing in graviceptive network, resulting in an abnormal deviation of perceived verticality. This can, in turn, align the longitudinal body axis with erroneous perceived verticality, consequently producing PS. Indeed, our PD-PS patients often tilted to the same direction of SVV.

We also identified the characteristics differentiating between PD-PS patients with ipsiversive SVV and those without ipsiversive SVV. PD-PS patients with ipsiversive SVV showed distinct properties, possibly supporting a primary role of verticality perception deficits in generation of PS. Even though postural balance was compromised in our PD-PS, consistent with a previous study^29^, PD-PS patients with ipsiversive SVV had more difficulty maintaining postural balance compared to those without ipsiversive SVV. This is likely due to primary adoption of altered verticality perception rather than compensation for progressive body inclination. In addition, more frequent sustained activity of paraspinal muscles contralateral to the leaning side might be interpreted as reflexive, but inefficient, muscle contraction to overcome postural instability. In contrast, PD-PS patients without ipsiversive SVV exhibited suggestive features of other underlying causes for PS. For instance, our PD-PS patients without ipsiversive SVV more frequently reported chronic back pain. Back pain may be attributable to postural deformities, by prompting protective posturing from further pain and restricting spinal motion^3,14^. Notably, this patient group maintained better postural balance than PD-PS patients with ipsiversive SVV, although the degree of SVV and PS were similar between groups. Presumably compensatory adjustment, rather than primary alteration, of verticality reference system might occur to restore postural balance after a body tilt from causative factors other than verticality misperception. Taken together, verticality misperception may be associated with PS in different pathomechanisms by participating in primary alteration or compensatory adjustment in central graviceptive pathway.

It has been also suggested that asymmetric functioning of basal ganglia circuit has a primary role in developing PS^1,2^. This hypothesis is corroborated by animal and clinical data demonstrating hemiparkinsonian animal models bending to the denervated striatum^30^, greater motor symptom asymmetry in PS^4^, and directionality of PS preferentially toward the less affected hemibody^5,31^. Nevertheless, neither asymmetry nor laterality of motor symptoms was associated with PS in our patients, consistent with a recent large study^14^. Furthermore, a recent autopsy report showed no pathological evidence of asymmetric involvement in a PD patient with PS^32^. These findings might reinforce the concept that multiple factors are involved in PS, apart from unbalanced dopaminergic functioning^1–3^.

Vestibular dysfunction has also been reported to have a critical role in generating PS in PD^31,33^. In a study evaluating vestibular function by bithermal caloric testing, all PD patients with PS exhibited unilateral vestibular hypofunction, exclusively in the same direction of PS^31^. In some of our PD patients, vestibular responses to caloric stimuli were decreased unilaterally. However, the frequency of unilateral CP was similar in both PD-PS and PD-noPS patients. These discordant findings may stem from differences in patients characteristics including relatively lesser degree of PS and the absence of spontaneous nystagmus, denoting long-standing compensation, in our patients. Instead, all PD-PS with unilateral CP tilted toward the same side of CP, regardless of patterns of SVV or motor symptoms laterality (see Supplementary Table). Together, it is assumable that unilateral vestibular hypofunction during caloric stimulation may play a part in PS, possibly by determining the direction of PS, toward the ipsilateral side of reduced caloric response. Future investigations addressing neural correlates for PS and unilateral CP in a larger sample of PD patients can further define the precise role of subclinical vestibular hypofunction in PS.

In favor of dystonic etiology, several EMG investigations have reported tonic activation of paraspinal or abdominal muscles ipsilateral to the leaning side in PD patients with PS^4–6,20,34^. However, this pattern was not observed in our PD-PS. Instead, our PD-PS patients usually presented sustained muscle activity contralateral to the leaning side, consistent with previous studies which interpreted this EMG pattern as muscle contraction compensating for body inclination^5,6^. These contradictory findings might reflect methodological variability, including muscles explored and EMG recording paradigm, or different patient characteristics, such as disease severity and duration of PS. Indeed, we did not specifically examine EMG activity of the abdominal muscles, such as the external oblique, which are a potential target of lidocaine injection therapy to ameliorate PS^34^. Nevertheless, there have been arguments against dystonic hypothesis, due to a paucity of clinical features compatible with dystonia in PS, including overflow, twisting or twitching, and aggravation with motion^1,2^. Furthermore, a recent series demonstrated that only a small number of PD patients with PS improved their postural deformities using sensory tricks that typically ameliorate dystonia^14^. Nevertheless, some PD patients with PS have reported beneficial effects of botulinum toxin or lidocaine injections, which relieve dystonic contraction of paraspinal or external oblique muscles^20,34,35^. Further investigations using a unified EMG protocol in a large population of PD patients with PS are required to clarify this issue.

Our study has several limitations. Firstly, the PD-noPS group contained fewer patients than the PD-PS group because patients in the two groups were matched for disease severity, which increased with the presence of PS^14^. Secondly, although global cognitive function did not differ between patient groups, we did not perform detailed assessments of executive or visuospatial dysfunction, which may also have a role in the development of PS^36,37^. Thirdly, healthy controls or patients with PS due to other diseases were not included in our study. Fourthly, as the vestibular measurements used in our study assess the angular VOR or utricular pathway, possible involvement of vestibular system other than these two pathways might have been missed in our PD patients with PS. Finally, cross-sectional nature of this study did not allow clarification regarding the causal relationship between abnormal verticality perception and PS in PD patients. For instance, different features between PD-PS patients with ipsiversive SVV and those without may be interpreted as the dynamic evolution of the SVV tilt from reactive to the postural misalignment to compensatory to postural imbalance. Longitudinal monitoring of verticality perception, plus other potential contributors, can help verify whether PS is a primary pathology of PS, compensatory adaptation for postural imbalance, or a reactive change to the postural misalignment elicited by other factors, such as CP.

To conclude, verticality misperception can be a strong risk factor for the generation of PS in PD patients. Especially, our study provides discriminating clinical profiles which can imply the primary alteration of verticality perception in PD patients with PS who tilt toward the perceived verticality or compensatory deviation in those who do not. This result may highlight differing roles of verticality perception in pathogenesis of PS depending on the congruence between the direction of perceived verticality and that of PS. Quantitative assessment of verticality perception deficits in PD patients with PS may increase our understanding of pathomechanisms of PS. Future prospective studies investigating verticality perception deficits in PD patients might provide quantitative measures predicting a high-risk population of PS. Moreover, these will help develop new therapeutic strategies manipulating verticality perception in PD patients with PS and define suitable patients for those therapies for alleviating PS.

## Electronic supplementary material

Supplementary Information
