# Supplementary material for: Pisa Syndrome in Parkinson’s Disease: Pathogenic Roles of Verticality Perception Deficits
Source: Sci Rep. 2018 Jan 29;8:1804. doi: 10.1038/s41598-018-20129-2 (PMC5788854; doi:10.1038/s41598-018-20129-2)
Supplement: Supplementary file 1 — Supplementary Information [file 41598_2018_20129_MOESM1_ESM.pdf]

Pisa Syndrome in Parkinson's Disease: Pathogenic Roles of Verticality Perception Deficits

Young Eun Huh[1], Kunhyun Kim[2], Won-Ho Chung[3], Jinyoung Youn[2], Seonwoo Kim[4], Jin Whan Cho[2,*]

[1]Department of Neurology, CHA Bundang Medical Center, CHA University, Seongnam, 13496, Korea
[2]Department of Neurology, Sungkyunkwan University School of Medicine, Samsung Medical Center, Seoul, 06351, Korea
[3]Department of Otolaryngology, Head and Neck Surgery, Sungkyunkwan University School of Medicine, Samsung Medical Center, Seoul, 06351, Korea
[4]Statistics and Data Center, Samsung Medical Center, Seoul, 06351, Korea
* email: jinwhan.cho@samsung.com

Supplementary information: Supplementary Table

	**PD-PS with unilateral CP (n=8)**
**Age (years)**	66.0 (53.8-78.3)
**Male gender (%)**	3 (37.5)
**Disease duration (years)**	14.0 (4.8-23.3)
**Hoehn and Yahr stage**	2.5 (2-3)
**UPDRS-III**	24.5 (12.0-37.0)
**PD motor subtype (PIGD/TD)**	4/4
**Degree of PS (°)**	14.0 (10-18)
**Direction of PS (R/L)**	4 (50.0)
**Abnormal SVV (%)**	6 (75.0)
**Direction of SVV (R/L)**	1/5
**Ipsiversive SVV**	4
**Asymmetry of motor symptoms**	4.0 (0.2-7.8)
**PS tilting to the less affected hemibody (%)**	2 (25.0)
**Dominant side of motor symptoms (R/L)**	4/4
**CP (%)**	8 (100.0)
**Side of CP (R/L)**	4/4
**Ipsilateral CP**	8
**EMG patterns of paraspinal muscles (%)**	
**Contralateral hyperactivity**	3 (37.5)
**Bilateral hyperactivity**	5 (62.5)
**Back pain (%)**	3 (37.5)
**BBS**	47.0 (45.2-48.8)
**LEDD (mg)**	688.0 (499.2-876.8)
**Treatment regimen (%)**	
**Levodopa + dopamine agonist**	7 (87.5)
**Levodopa**	0
**Dopamine agonist**	1 (12.5)
**BMI (kg/m^2)**	24.8 (20.3-29.3)
**MMSE**	27.5 (25.5-29.5)
**Education (years)**	16.0 (7.5-24.5)

Supplementary table. Demographic and clinical measurements in PD-PS with unilateral CP. Data are presented as median (interquartile range) unless otherwise noted. BBS: Berg Balance Scale, BMI: body mass index, CP: canal paresis, LEDD: levodopa equivalent daily dose, MMSE: Mini-Mental State Examination, PIGD: postural instability and gait disturbance, SVV: subjective visual vertical, TD: tremor dominant, UPDRS-III: Unified Parkinson's Disease Rating Scale motor score.
